# Effects of Unilateral Leg Training Using Elastic and Inelastic Barbells on Dynamic Balance and Muscle Activation: A Randomized Controlled Trial

**DOI:** 10.3390/sports14100450

**Published:** 2026-10-12

**Authors:** Jihwan Jeong, Jayeon Lee, Chaekwan Lee, Shuho Kang, Ilbong Park

**Affiliations:** 1 Department of Convergence Sports Instruction, Hwashin Cyber University, Busan 47558, Republic of Korea; 2 Department of Sports Health Convergence, Busan University of Foreign Studies, Busan 46234, Republic of Korea; 20236202@bufs.ac.kr (J.L.); 20246001@bufs.ac.kr (C.L.); 20236204@bufs.ac.kr (S.K.); fnjboss@bufs.ac.kr (I.P.)

**Keywords:** elastic barbell, unilateral leg training, single-leg deadlift, dynamic balance, center of pressure, electromyography, perturbation-based training, muscle activation

## Abstract

Background: Elastic barbells generate continuous, unpredictable perturbations through elastic deformation of the bar, delivering a top-down instability stimulus that transfers from the upper to the lower limbs. Whether repeated exposure produces training-related changes in muscle activation, and whether any postural control gains transfer to an untrained movement condition, remains unknown. Methods: Twenty-eight resistance-trained men (mean age 22.8 ± 1.6 years; experimental group (EG) 23.4 ± 1.4, control group (CG) 22.3 ± 1.7) were randomly assigned to train with an elastic barbell (EG, *n* = 14) or an inelastic barbell (CG, *n* = 14). Both completed an identical progressive unilateral leg training program twice weekly for 12 weeks (20–40% of one-repetition maximum). Pre- and post-intervention measures were the Y-Balance Test (YBT), centre of pressure (COP) displacement during single-leg deadlift (SLDL), and surface electromyography (%RVC) of seven trunk and lower-limb muscles across three lifting speeds. Data were analyzed with mixed-design ANOVA (α = 0.05). Results: The EG showed greater gains in dynamic balance; the post-intervention YBT composite score was significantly higher in the EG (102.55 ± 6.22%) than in the CG (90.65 ± 12.95%; Group × Time *F*(1, 26) = 7.25, *p* = 0.012). A significant Group × Time interaction for COP under the untrained inelastic barbell task (anterior–posterior *F*(1, 26) = 8.86, *p* = 0.006; medial–lateral *F*(1, 26) = 5.61, *p* = 0.026) indicated task-specific transfer of postural control. The semitendinosus was the only muscle with a significant Group × Time interaction in both barbell tasks, with activation decreasing in the EG and increasing in the CG. Conclusions: Elastic barbell training improved dynamic balance and altered lower-limb muscle activation more than inelastic barbell training, and the postural control benefit transferred to the same task performed with an untrained barbell. Because injury outcomes and motor-unit behaviour were not assessed, injury outcomes were not assessed; any application to injury prevention remains a hypothesis for future investigation.

## 1. Introduction

Dynamic stability during single-limb support is a decisive determinant of performance in athletic tasks such as acceleration, deceleration, landing, and change of direction, and it is directly linked to the risk of non-contact lower-limb injury [1,2]. Identifying training methods that effectively enhance this capacity therefore remains a continuing objective in sports science and rehabilitation. Among unilateral exercises, the single-leg deadlift (SLDL) is widely used because, as a closed-kinetic-chain movement, it emphasizes neuromuscular coordination of the posterior chain over isolated strength development [3,4,5]. The posterior chain and hamstring activation patterns elicited by this exercise have been proposed as relevant to posterior chain conditioning, although its direct effect on injury incidence has not been established.

Conventional strength and balance training is typically performed in predictable environments using repetitive, controlled stimuli [6,7]. Such approaches, however, insufficiently reproduce the unpredictable external disturbances—impacts and abrupt directional changes—that occur in competition [1]. To address this limitation, instability-based resistance training has attracted attention. Although instability tools were traditionally discouraged for pure strength gains [8], recent evidence indicates that instability-augmented resistance exercise can enhance the neuromuscular system more than conventional loading by promoting activation of stabilizing musculature in unstable environments [9,10,11,12].

Most instability research, however, has concentrated on bottom-up approaches, in which instability is introduced at the base of support (e.g., BOSU, wobble boards) and transmitted from the lower to the upper limbs. In contrast, top-down instability—originating in the upper limbs and propagating distally through the kinetic chain—has been examined far less, and its longitudinal effects remain poorly defined [13,14]. A representative top-down tool is the elastic barbell: a flexible, plastic-composite bar whose elastic deformation generates vertical and lateral oscillations during movement, delivering continuous, unpredictable perturbations proposed to enhance joint stabilization and coordination [15,16].

Recent work has begun to characterize the acute response to elastic barbells. In American-football players, squats performed with a flexible versus a steel barbell produced significant differences in lower-limb electromyography (EMG) and ground-reaction forces [17]. Our prior cross-sectional study further demonstrated that, relative to an inelastic barbell, the elastic barbell reduced COP displacement and increased gluteus medius, hamstring, and gastrocnemius activation during the SLDL, especially at higher speeds [18]. Nevertheless, a single-session design cannot determine whether these acute responses persist or change after a period of training, because the response to a transient stimulus and the adaptation learned through repeated exposure are governed by fundamentally different mechanisms [19,20].

Two questions therefore remain open. First, do the acute advantages of elastic barbell exercise consolidate into chronic adaptations after a structured training period? Second, does perturbation-based training generalize—that is, does postural control acquired with the elastic barbell transfer to an untrained, inelastic movement environment? Generalization of this kind is difficult to detect with cross-sectional or short-term designs and, to our knowledge, has not been tested for a top-down instability tool.

The Y-Balance Test was selected as a primary functional outcome because it is a reliable, validated field measure of dynamic single-limb postural control, and its anterior, posteromedial, and posterolateral reach directions load the hip stabilizers and posterior chain that the SLDL trains; it therefore provides a functional index of whether training-induced control transfers beyond the trained task. Because both neuromuscular demand and the amplitude of elastic bar oscillation increase with movement velocity, we further reasoned that any advantage of elastic barbell training would be expressed most clearly under higher-speed conditions.

Accordingly, this study investigated the effects of a 12-week unilateral leg training program performed with elastic versus inelastic barbells on dynamic balance (YBT), postural stability (COP), and trunk and lower-limb muscle activation (surface EMG) in healthy young men. Aligned with the two research questions above and with this velocity rationale, we hypothesized that, compared with inelastic barbell training, elastic barbell training would (i) produce greater improvements in Y-Balance performance (chronic adaptation), (ii) yield greater postural stability that transfers to the untrained inelastic task (task transfer), and (iii) produce a distinct pattern of muscle activation, with these effects expressed most strongly under higher-speed conditions.

## 2. Materials and Methods

### 2.1. Participants

Healthy men in their twenties with no musculoskeletal injury in the preceding year were recruited by public notice at the university. Eligibility required (i) age ≥ 20 years without musculoskeletal disease and (ii) at least one year of resistance training experience (≥two sessions per week; mean 3.2 ± 1.4 years of experience). Individuals with a history of lumbar, pelvic, or lower-limb disorders, or with upper-limb musculoskeletal pain or inflammation, were excluded. The a priori sample size was estimated in G*Power 3.1 (F-test family; ANOVA: repeated measures, within–between interaction) for the primary outcome (EMG), with an effect size f = 0.25, α = 0.05, and power = 0.80, based on prior SLDL comparisons [3,21,22]; this yielded a minimum of 24 participants, and 30 were enrolled to allow for a 15–20% dropout rate.

After baseline testing, the 30 participants were randomized 1:1 to the experimental group (EG; elastic barbell) or the control group (CG; inelastic barbell). Two participants (one per group) withdrew during the intervention because of mandatory military service, leaving 28 participants (EG, *n* = 14; CG, *n* = 14) for analysis. All participants used the right leg as the dominant limb. Baseline characteristics did not differ between groups (all *p* > 0.05; Table 1). The study was conducted in accordance with the Declaration of Helsinki and approved by the Public Institutional Review Board (approval no. P01-202604-01-014, date of approval 6 April 2026). All participants provided written informed consent after being fully informed about the aims and procedures of the study.

### 2.2. Study Design

This was a two-arm, parallel-group randomized controlled trial with a pre–post design. Group allocation was performed immediately after baseline testing using a computer-generated 1:1 sequence. Because of the visible physical differences between the barbells, blinding of participants and trainers was not feasible; however, data coding and statistical analysis were conducted by an independent investigator blinded to allocation. Baseline and post-intervention assessments comprised the Y-Balance Test and, during the SLDL, COP and surface EMG measured with both the elastic and inelastic barbells. Thus, every participant was evaluated under both barbell tasks at both time points, so that the inelastic barbell task served as an untrained transfer probe for the EG and the elastic barbell task as an untrained transfer probe for the CG. This trial was prospectively registered in the Clinical Research Information Service (CRIS; registration no. KCT0012036).

### 2.3. Training Program

The unilateral leg training program was structured according to Boyle’s functional-training framework and the dynamic-systems perspective of motor coordination [7,23]. Sessions were held twice weekly for 12 weeks (24 sessions); each 50 min session comprised a 10 min warm-up, 30 min of main training, and a 10 min cool-down. The EG used the elastic barbell and the CG the inelastic barbell for all main exercises, which were otherwise identical. The program was periodized into a neuromuscular control phase (weeks 1–4, 20% of one-repetition maximum [1RM]), a functional adaptation phase (weeks 5–8, 30% 1RM), and a power phase (weeks 9–12, 40% 1RM), with movement velocity and plyometric-box use progressively increased to raise task difficulty (Table 2).

For each main exercise, the 1RM was estimated once, one week before the intervention, from a 7–10-repetition submaximal test using a validated prediction equation [24]. The prescribed relative load (20%, 30%, and 40% 1RM in successive phases) was calculated from this baseline 1RM and was not re-estimated during the intervention. Each exercise was performed for 3 sets of 10 repetitions, with 45 s of rest between sets and approximately 1 min between exercises. Progression to the next movement velocity or plyometric-box height occurred once a participant completed all prescribed repetitions with correct technique. Concentric velocity progressed from controlled (weeks 1–4) to increased (weeks 5–8) to maximal-intent (weeks 9–12); velocity was cued by a metronome and by verbal instruction but was not instrumented. All 28 participants completed the full program (24 of 24 sessions; 100% adherence); any session that could not be attended on the scheduled day was made up within the same week.

### 2.4. Measurements and Procedures

#### 2.4.1. Y-Balance Test

Dynamic balance was assessed with the Y-Balance Test kit (Functional Movement Inc., Chatham, VA, USA). Standing on the dominant limb, participants reached with the non-dominant limb in the anterior (ANT), posteromedial (PM), and posterolateral (PL) directions. Following the protocol of Plisky et al. [25], six practice and three recorded trials were performed per direction, with 5 s of rest between trials; the mean reach distance (cm) was retained. Limb length was measured (cm) as the distance from the anterior superior iliac spine to the distal tip of the medial malleolus with the participant supine. For each participant, the composite score (%) was computed as (ANT + PM + PL)/(3 × limb length) × 100, and group values represent the mean of these individual scores.

#### 2.4.2. Single-Leg Deadlift Task, COP, and Surface EMG

**Figure 1:**
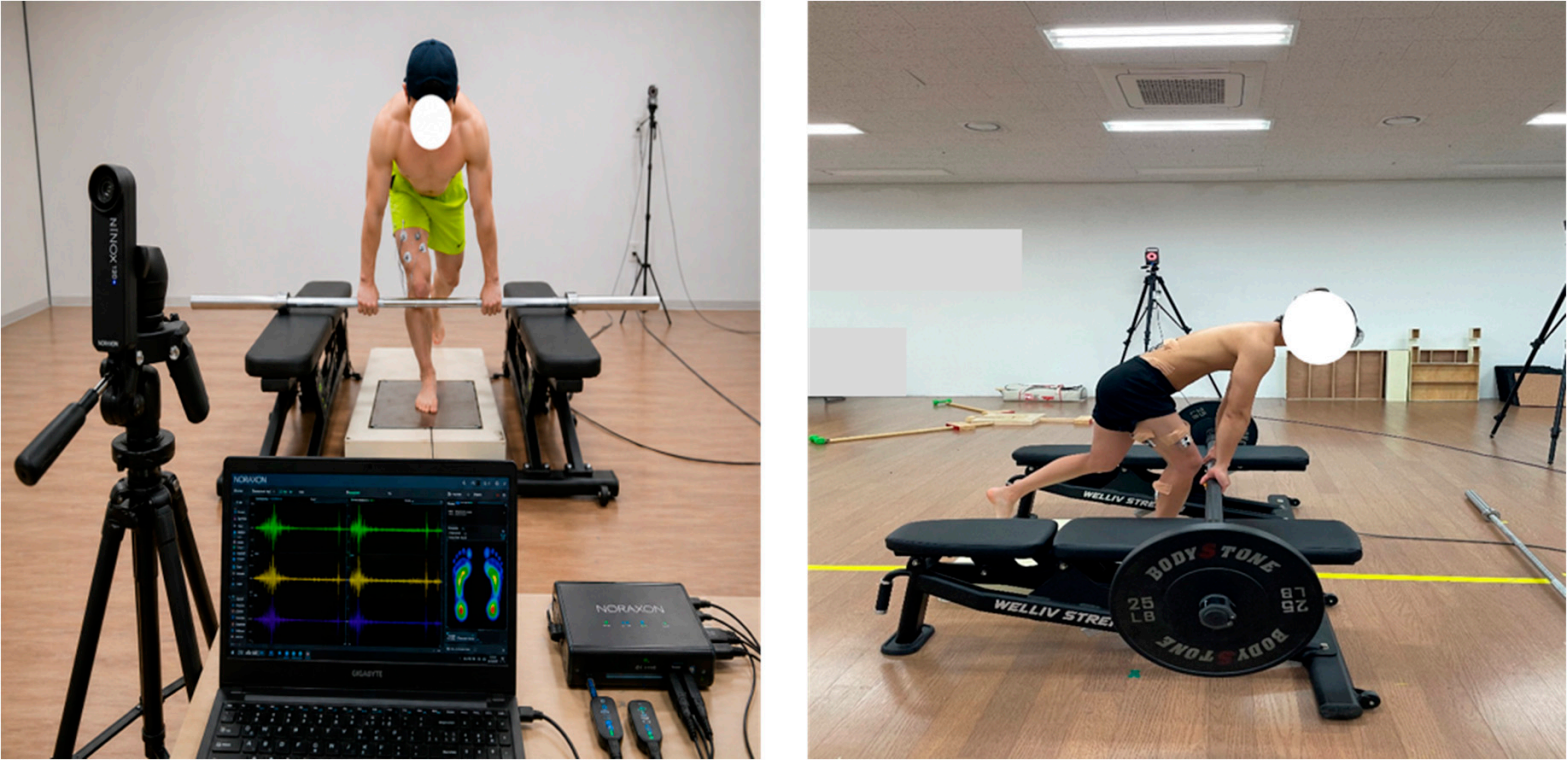
The subject performed a single-leg deadlift movement under both conditions.

During the SLDL, COP and surface EMG were recorded simultaneously with both barbells. The elastic barbell (Stabibar, Smartfit, Busan, Republic of Korea) was a flexible composite bar with an overall length of 2.2 m and a mass of 3 kg; the inelastic barbell was a standard Olympic barbell (2.2 m, 20 kg). The load was set to 30% of the SLDL 1RM [17], with the 1RM estimated one week earlier from a 7–10-repetition submaximal test for safety [24]. The analysis window extended from lift-off to a 2 s hold at full hip and knee extension [18]. Each barbell was lifted at three velocities—normal (2 s), fast (1 s), and power (maximal intent)—in randomized barbell order, with three trials per condition paced by a metronome. Rest of ≤5 min separated sets; trials with balance loss or foot displacement were repeated after 1 min. A 2D video system (NiNOX 120, Noraxon Inc., Scottsdale, AZ, USA) synchronized the force plate and EMG signals (Figure 1).

COP was recorded with a force plate (AMTI-OR6, Watertown, MA, USA) at 1000 Hz; a 10-N threshold defined valid data, non-slip tape was applied, and equipment was pre-warmed ≥45 min to minimize electrical noise [26]. Anterior–posterior (AP) and medial–lateral (ML) displacement ranges were computed in Nexus v2.15 (Vicon, Oxford, UK); smaller values indicate greater postural stability.

Surface EMG (Ultium system, Noraxon Inc., Scottsdale, AZ, USA) was recorded from seven muscles on the dominant side—right and left erector spinae (Rt. ES, Lt. ES), gluteus medius (GM), biceps femoris (BF), semitendinosus (ST), vastus lateralis (VL), and vastus medialis (VM)—with Ag/AgCl electrodes placed per SENIAM guidelines after skin preparation. Signals were sampled at 2000 Hz, band-pass-filtered (20–400 Hz), and processed with a 150 ms root-mean-square window. To ensure between-session reproducibility in this longitudinal design, activation was normalized to a reference voluntary contraction (%RVC)—a standardized submaximal SLDL performed with a 10 kg inelastic barbell at normal (2 s) speed—because maximal-voluntary-contraction normalization is itself susceptible to training-induced change and reduced reproducibility over time [27,28,29].

### 2.5. Statistical Analysis

Data were analyzed in SPSS 28.0 (IBM Corp., Armonk, NY, USA) and are presented as mean ± SD. Normality was assessed with the Shapiro–Wilk test and baseline homogeneity with independent *t*-tests. Intervention effects were examined with mixed-design (Group × Time) analysis of variance; for COP and EMG, three-way (Group × Time × Speed) mixed-design ANOVA was used because velocity comprised three levels. Where Mauchly’s test indicated a violation of sphericity for the speed factor, Greenhouse–Geisser-corrected degrees of freedom were applied. Within-group changes were tested with paired *t*-tests, between-group post-intervention differences with independent *t*-tests, and pairwise comparisons with Bonferroni correction. Results are reported as F(df1, df2) with exact *p*-values, and partial eta-squared (η^2^p) indexed effect size; significance was set at α = 0.05.

## 3. Results

### 3.1. Dynamic Balance (Y-Balance Test)

Both groups improved in dynamic balance after the intervention, but the pattern differed by barbell type (Table 3). In the ANT direction, both groups improved significantly (Time *F*(1, 26) = 55.66, *p* < 0.001, η^2^p = 0.682) with no Group × Time interaction, consistent with a general effect of unilateral loading. In the PM direction, a significant Group × Time interaction (*F*(1, 26) = 6.24, *p* = 0.019, η^2^p = 0.194) accompanied the time effect, and the EG exceeded the CG post-intervention (t = 2.49, *p* = 0.020). For the PL direction, both groups improved over time (Time *F*(1, 26) = 63.78, *p* < 0.001, η^2^p = 0.710) and a group main effect was present (*F*(1, 26) = 5.82, *p* = 0.023, η^2^p = 0.183); however, the Group × Time interaction did not reach significance (*F*(1, 26) = 4.11, *p* = 0.053), so a differential training effect in this direction is not claimed.

**Figure 2:**
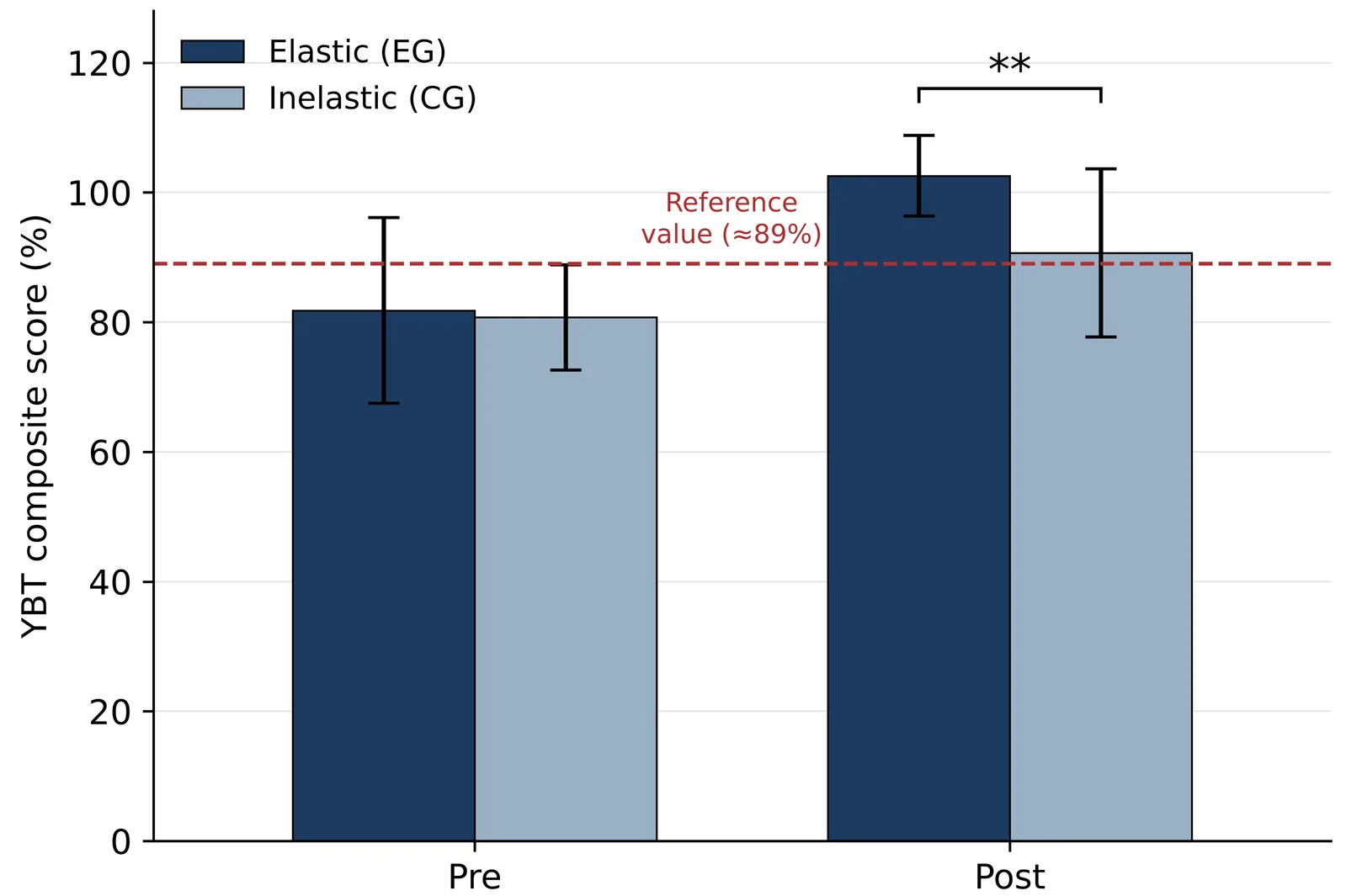
Y-Balance Test composite score before and after the 12-week intervention in the elastic-barbell group (EG) and the inelastic-barbell group (CG). Vertical error bars denote the standard deviation (SD). The dashed line marks a YBT composite reference value (≈89%) reported in prior cohorts and is shown for descriptive comparison only. The horizontal bracket indicates a significant post-intervention difference between groups (** *p* < 0.01, EG vs. CG).

The composite score, a key balance outcome, showed a significant Group × Time interaction (*F*(1, 26) = 7.25, *p* = 0.012, η^2^p = 0.218). Post-intervention, the EG (102.55 ± 6.22%) was significantly higher than the CG (90.65 ± 12.95%; t = 3.10, *p* = 0.005); the EG mean was also above a YBT composite reference value (≈89–90%) reported in prior cohorts as associated with higher lower-limb injury risk, whereas the CG mean was close to it [25] (Figure 2). This reference value is presented descriptively and not as a validated clinical threshold for the present sample.

### 3.2. Postural Stability (Centre of Pressure)

COP displacement decreased in both groups, but the direction- and task-specific patterns produced the study’s central findings (Table 4). Under the elastic barbell task, AP displacement improved across time and speed (Time *F*(1, 26) = 44.43, *p* < 0.001, η^2^p = 0.631; Speed *F*(2, 52) = 16.27, *p* < 0.001), with the EG lower than the CG post-intervention at normal (t = −2.62, *p* = 0.015) and power (t = −5.78, *p* < 0.001) speeds; ML displacement likewise decreased (Time *F*(1, 26) = 32.79, *p* < 0.001, η^2^p = 0.558), with the EG lower at power speed (t = −4.13, *p* < 0.001).

**Figure 3:**
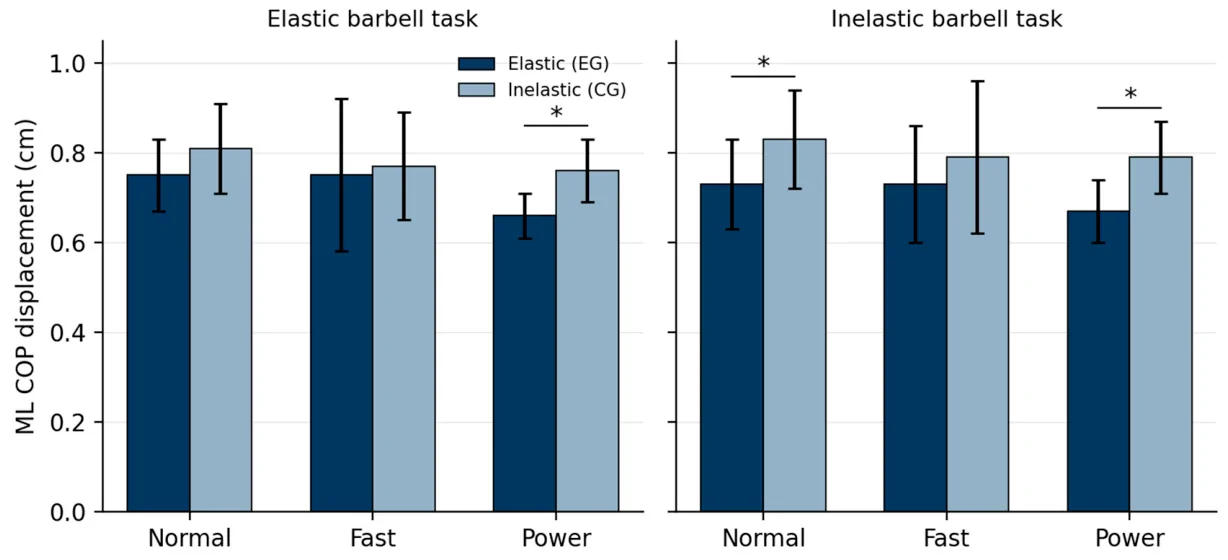
Post-intervention medial–lateral (ML) center-of-pressure (COP) displacement during the single-leg deadlift across speeds. The elastic-barbell group (EG) was more stable than the inelastic-barbell group (CG) under both the trained elastic task and the untrained inelastic task, illustrating a transfer effect. Vertical error bars denote the standard deviation (SD); horizontal brackets indicate significant post-intervention differences between groups (* *p* < 0.05, EG vs. CG).

The key result emerged under the untrained inelastic barbell task. Despite the EG never having trained with the inelastic barbell, a significant Group × Time interaction appeared for AP displacement (*F*(1, 26) = 8.86, *p* = 0.006, η^2^p = 0.254) with a Speed × Time interaction (*F*(2, 52) = 6.17, *p* = 0.007), and the EG was significantly more stable than the CG post-intervention at normal (t = −2.45, *p* = 0.022) and power (t = −4.85, *p* < 0.001) speeds. ML displacement showed the same pattern (Group × Time *F*(1, 26) = 5.61, *p* = 0.026, η^2^p = 0.177; EG < CG at normal, t = −2.50, *p* = 0.019, and power, t = −4.21, *p* < 0.001). Thus, postural control acquired with the elastic barbell transferred to an untrained movement environment (Figure 3).

Consistent with these interactions, a between-group difference was present post-intervention in the ML direction (*F*(1, 26) = 8.62, *p* = 0.007, η^2^p = 0.249), the largest group effect among the postural stability analyses. Because the pre- and post-intervention partial eta-squared (η^2^p) values for the barbell effect were estimated in separate models, they were not compared statistically to infer a change in the barbell effect over time.

### 3.3. Neuromuscular Activation (Surface EMG)

Across all seven muscles, the movement speed factor was the dominant source of variance, whereas barbell type modified the adaptation of only one muscle. The results are summarized in Table 5.

**Figure 4:**
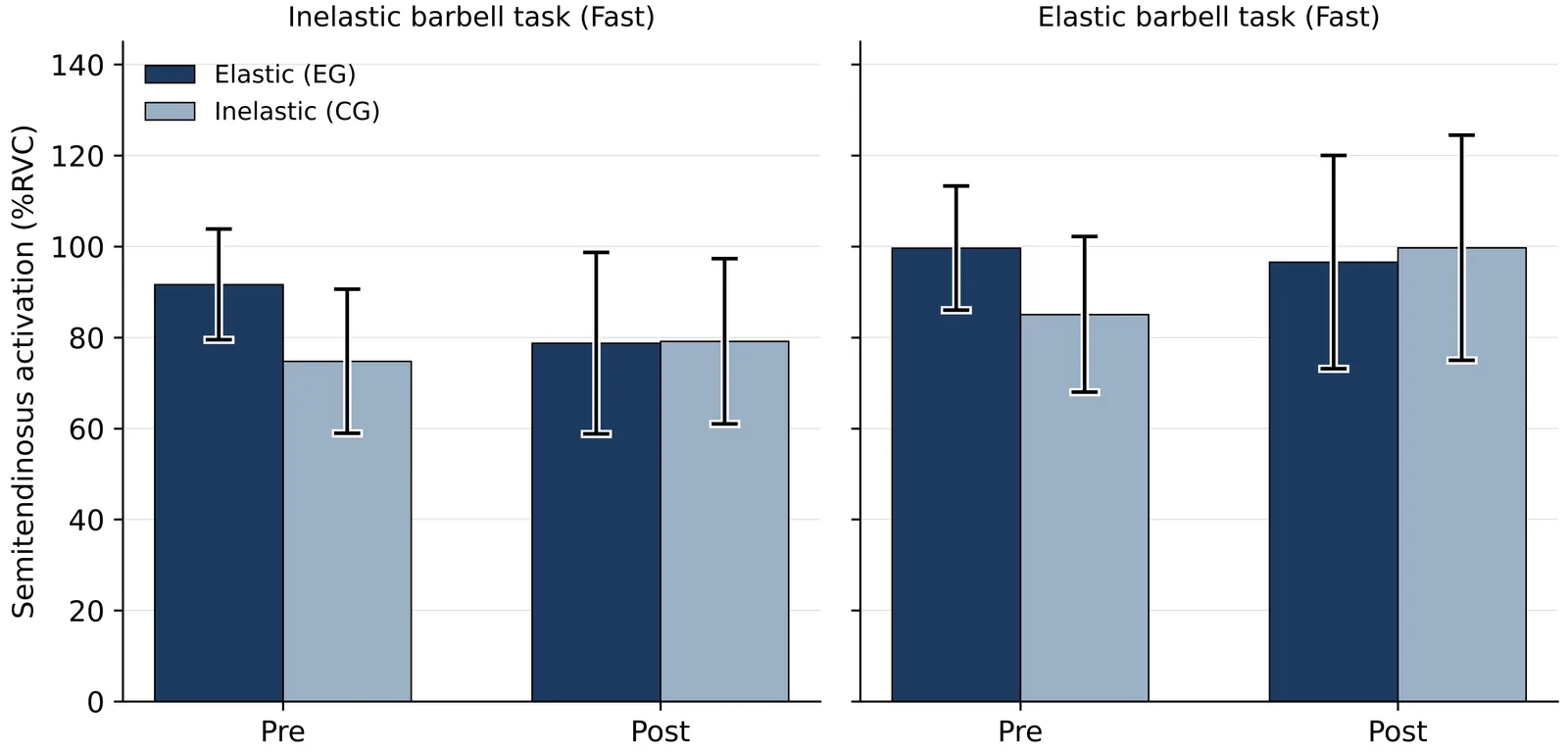
Semitendinosus (ST) activation, expressed as a percentage of the reference voluntary contraction (%RVC), at fast speed before and after the intervention. Activation decreased in the elastic-barbell group (EG) and increased in the inelastic-barbell group (CG) at a matched relative load. Vertical error bars denote the standard deviation (SD).

The semitendinosus (ST) was the only muscle to show a significant Group × Time interaction under both barbell tasks (inelastic: *F*(1, 26) = 4.77, *p* = 0.038, η^2^p = 0.155; elastic: *F*(1, 26) = 4.36, *p* = 0.047, η^2^p = 0.144), and the two groups changed in opposite directions. In the EG, ST activation for the same relative load decreased from pre- to post-intervention (inelastic task: 95.88 → 81.18 %RVC at normal speed and 91.67 → 78.73 %RVC at fast speed; both *p* < 0.05), whereas in the CG, it increased (inelastic task: 80.15 → 96.46 %RVC at power speed; elastic task: 85.09 → 99.72 %RVC at fast speed and 93.86 → 124.62 %RVC at power speed; all *p* < 0.05). A significant Speed × Time interaction (*F*(2, 52) = 5.29, *p* = 0.012, η^2^p = 0.297) and Speed main effect (*F*(2, 52) = 11.60–12.19, *p* < 0.001) accompanied these changes, whereas the Time main effect was non-significant (Figure 4).

For the biceps femoris (BF), a significant Time main effect emerged under the elastic task only (*F*(1, 26) = 7.30, *p* = 0.012, η^2^p = 0.219), reflecting an increase in activation from pre- to post-intervention in both groups, with no Group × Time interaction. The gluteus medius (GM) was governed by movement speed, showing a large Speed main effect (inelastic: *F*(2, 52) = 14.00, *p* < 0.001, η^2^p = 0.528; elastic: *F*(2, 52) = 13.82, *p* < 0.001, η^2^p = 0.525) together with a Speed × Time interaction (inelastic: *F*(2, 52) = 3.73, *p* = 0.038; elastic: *F*(2, 52) = 3.77, *p* = 0.037) but no Group × Time interaction. The erector spinae likewise showed speed-dependent activation; the left erector spinae (Lt. ES, contralateral to the right-dominant supporting limb) produced the largest Speed × Time interaction of the analysis under the inelastic task (*F*(2, 52) = 7.07, *p* = 0.004, η^2^p = 0.361), while the right erector spinae (Rt. ES) showed a strong Speed main effect (elastic: *F*(2, 52) = 22.59, *p* < 0.001, η^2^p = 0.644).

The knee extensors were driven by speed alone. The vastus lateralis (VL) and vastus medialis (VM) showed very large Speed main effects (VL: *F*(2, 52) = 23.86 [inelastic] and 43.93 [elastic]; VM: *F*(2, 52) = 22.70 [inelastic] and 34.32 [elastic]; all *p* < 0.001, η^2^p = 0.645–0.778) but no Group × Time interaction under either task, indicating that quadriceps activation was determined by movement velocity and was unaffected by barbell type; the VM Group main effect approached significance under the elastic task only (*F*(1, 26) = 4.02, *p* = 0.055).

## 4. Discussion

This randomized controlled trial compared 12 weeks of unilateral leg training with elastic versus inelastic barbells and yielded three principal findings: elastic barbell training produced (i) greater improvements in dynamic balance, (ii) greater gains in postural stability that transferred to the same SLDL performed with an untrained (inelastic) barbell—that is, task-specific transfer—and (iii) a between-group difference in semitendinosus activation. These results extend our prior acute observations [18] to a 12-week training context; because injury outcomes and motor-unit behaviour were not measured, mechanistic and injury prevention interpretations are offered as hypotheses rather than established effects.

### 4.1. Dynamic Balance and Sensorimotor Integration

Although both groups improved in Y-Balance performance, significant Group × Time interactions—indicating greater gains with the elastic barbell—were found for the posteromedial reach and the composite score, whereas the posterolateral reach showed a group main effect and a time effect without a significant interaction. ANT reach depends largely on quadriceps contraction and ankle plantar-flexion mobility, which are sufficiently stimulated by repetitive unilateral loading regardless of instability; the absence of a between-group difference there reflects a general training effect. In contrast, PM and PL reach are limited primarily by the eccentric control and stability provided by the hip extensors, hip abductors and external rotators, and the pelvis-stabilizing trunk musculature [30,31]—the muscles that must resist the multi-planar oscillation of the elastic bar during single-limb support. We therefore propose that repeated exposure to these micro-perturbations preferentially trained frontal- and transverse-plane hip control, which is expressed more in the posteromedial and posterolateral reaches than in the sagittal-plane-dominant anterior reach. These effects are consistent with sensory reweighting, whereby repeated exposure to unpredictable perturbation may increase the weighting of proprioceptive input and refine cortical motor programs beyond spinal reflex adaptation [32,33].

### 4.2. Postural Control and the Transfer Effect

The COP findings constitute the study’s core contribution. Improvements under the trained elastic task grew larger as speed increased, in agreement with the principle that neural adaptation raises the rate of force development and is most evident in fast movements, and with the notion that vibration-like stimuli may heighten muscle-spindle sensitivity and motor-neuron excitability [20,34]. Both groups improved under both barbell tasks over the 12 weeks, consistent with a general training effect (significant time effects; Table 4). Crucially, however, a significant Group × Time interaction emerged only under the untrained inelastic task, where the EG improved more than the CG despite never training with that barbell. Thus, the additional postural control benefit conferred by elastic barbell training transferred to the same task performed with an untrained barbell, whereas the CG showed no comparable between-group advantage in its own untrained (elastic) task. Because both tasks were the same SLDL performed with a different barbell, this is best described as task-specific transfer rather than broad generalization [35,36], consistent with evidence that the transfer of perturbation-based training depends on the similarity between the trained and tested tasks [37].

The direction-specific pattern is also informative: the transfer interaction was significant in both the AP and ML directions, and in the ML direction a between-group difference was present post-intervention. Because AP control relies largely on a single-axis ankle strategy whereas ML control requires multi-joint hip-strategy coordination [38], the ML improvement suggests that elastic barbell training influenced frontal-plane hip control during this task. Reduced ML stability has been associated with lower-limb injury risk in previous work [1,39]; however, because injury incidence and joint loading were not assessed here, whether the present ML changes translate to altered injury risk cannot be determined and remains to be tested.

### 4.3. Divergent Semitendinosus Activation

The two groups changed semitendinosus (ST) activation in opposite directions. The EG’s lower ST activation at a matched relative load is consistent with improved neuromuscular efficiency. However, surface EMG expressed as RMS %RVC cannot resolve motor-unit recruitment or firing behaviour; refined motor-unit recruitment, altered rate coding, and reduced neural drive are therefore offered as possible explanations rather than demonstrated mechanisms, and confirming them would require decomposition-based or high-density EMG. The CG’s higher activation is consistent with an alternative strategy in which a greater quantity of activation is used to meet the progressive load [19,40]. The ST is well suited to expose such a divergence because, as the medial hamstring, it generates knee-flexion and internal-rotation moments and contributes to restraining anterior tibial translation, a role relevant to knee stability [41,42]; whether the observed activation difference confers any protective effect was not tested. That the medial hamstring diverged between groups while the lateral BF increased in both groups may indicate branch-specific hamstring behaviour, which warrants further study.

The remaining muscles clarify what is general versus tool-specific. GM activation rose sharply at higher speeds in both groups, consistent with unilateral training’s established efficacy for hip-stabilizer engagement [43,44]. The large Speed × Time effect in the contralateral erector spinae highlights the trunk’s role in kinetic-chain force transmission during single-limb support [45,46]. The knee extensors, driven by velocity rather than barbell type, showed no differential change, implying that quadriceps activation was comparable between tools and that the differential effect of the elastic barbell was concentrated in stabilizing musculature such as the ST.

### 4.4. Methodological Considerations, Limitations, and Applications

Two methodological features strengthen the internal validity of these longitudinal comparisons: normalization to a standardized submaximal reference contraction (%RVC), which avoids the reproducibility problems of maximal-contraction normalization in training studies [27,28,29], and the inclusion of bilateral trunk muscles, which allowed kinetic-chain activation to be examined beyond the lower limb. Several limitations nonetheless apply. First, the sample comprised healthy young men, limiting generalization to women, adolescents, older adults, and injured populations. Second, although all participants met the same minimum resistance training criterion, their prior training backgrounds were not otherwise standardized; heterogeneous training histories may have contributed to the large between-participant variability observed for some outcomes (notably posteromedial reach). Third, movement velocity during training was prescribed and cued but not instrumented, and the physical magnitude of the perturbation was not measured; future studies should quantify concentric velocity and oscillation amplitude/frequency (e.g., with a linear position transducer or inertial measurement units) and relate them to muscle activation. Finally, the 12-week intervention did not assess injury outcomes, joint loading, or long-term retention.

Practical applications. A moderate-load (20–40% 1RM) unilateral leg training program performed twice weekly for 12 weeks improved dynamic balance and single-leg deadlift postural control in resistance-trained men. Adding elastic barbell instability further increased medial–lateral postural demand, most clearly at maximal movement velocity, and produced postural control improvements that carried over to the same task with a standard barbell. Practitioners may therefore use elastic barbell SLDL variations, prescribed at higher velocities, as a low-cost way to progressively overload frontal-plane single-limb control once basic technique is established; standard barbell training remains sufficient when the goal is general strength or when instability is contraindicated. Because injury outcomes were not measured, any injury prevention use should be regarded as a hypothesis for future testing across different loading or perturbation conditions.

## 5. Conclusions

Twelve weeks of moderate-load unilateral leg training improved dynamic balance and single-leg deadlift performance in both groups. Training with an elastic barbell produced larger gains in dynamic balance and a greater improvement in postural stability that transferred to the same task performed with an untrained inelastic barbell, and it was associated with lower semitendinosus activation at a matched load. Because injury incidence and joint loading were not assessed, these findings do not address injury prevention; whether elastic barbell training reduces injury risk remains a hypothesis to be tested in future studies that directly measure injury incidence or joint loading. Practically, elastic barbell training may be a useful option when the goal is to challenge dynamic stability across different loading or perturbation conditions; its transfer to other tasks and surfaces was not tested and warrants further study.

## Figures and Tables

**Table 1:** General characteristics of the participants (mean ± SD).

Group	Age (Years)	Height (cm)	Weight (kg)
EG (*n* = 14)	23.36 ± 1.39	175.09 ± 5.78	74.06 ± 9.57
CG (*n* = 14)	22.29 ± 1.73	173.84 ± 6.39	77.10 ± 12.89

Note: EG, experimental group (elastic barbell); CG, control group (inelastic barbell). No between-group differences were observed (*p* > 0.05).

**Table 2:** Twelve-week unilateral leg training program.

Component	Weeks 1–4	Weeks 5–8	Weeks 9–12
Phase	Neuromuscular control	Functional adaptation	Power
Intensity	20% 1RM	30% 1RM	40% 1RM
Warm-up (10 min)	Lower-extremity mobility and dynamic stretching	Lower-extremity mobility and dynamic stretching	Lower-extremity mobility and dynamic stretching
Main (3 × 10; 30 min)	Split squat; lunge; step-up (plyo box); single-leg deadlift; single-leg squat; single-leg extension (plyo box); single-leg snatch	Same exercises; increased velocity and plyometric-box use	Same exercises; maximal-intent velocity and plyometric-box use
Cool-down (10 min)	Self-myofascial release and stretching	Self-myofascial release and stretching	Self-myofascial release and stretching

Note: 1RM, one-repetition maximum. The two groups performed identical exercises and volume, differing only in barbell type.

**Table 3:** Y-Balance Test reach distances and composite score (mean ± SD).

Variable	Group	Pre	Post	Time *F*(1, 26)	G × T *F*(1, 26)	η^2^p
ANT (cm)	EG	46.67 ± 6.66	55.36 ± 6.81	55.66 ***	0.15	0.682
	CG	48.27 ± 6.54	56.09 ± 10.08			
PM (cm)	EG	97.79 ± 24.33	126.83 ± 9.09	17.59 ***	6.24 *	0.404
	CG	98.34 ± 12.02	105.70 ± 30.45			
PL (cm)	EG	100.91 ± 18.44	125.46 ± 7.98	63.78 ***	4.11	0.710
	CG	95.54 ± 11.82	110.16 ± 11.77			
Composite (%)	EG	81.79 ± 14.32	102.55 ± 6.22	58.20 ***	7.25 *	0.691
	CG	80.72 ± 8.13	90.65 ± 12.95			

Note: ANT, anterior; PM, posteromedial; PL, posterolateral; G × T, Group × Time; η^2^p, partial eta-squared. Degrees of freedom were (1, 26) for all effects shown. Post-intervention EG > CG for PM (t = 2.49, *p* = 0.020), PL (t = 4.03, *p* < 0.001), and composite (t = 3.10, *p* = 0.005). * *p* < 0.05, *** *p* < 0.001.

**Table 4:** COP displacement (cm) during the single-leg deadlift by barbell task, direction, speed, and group (mean ± SD).

Task/Dir.	Speed	Group	Pre	Post	Effect (F, η^2^p)	Post Hoc
Elastic–AP	Normal	EG	1.17 ± 0.17	0.98 ± 0.14	Time *F*(1, 26) = 44.43 *** η^2^p = 0.631	EG < CG *
		CG	1.14 ± 0.14	1.15 ± 0.17		
	Fast	EG	1.10 ± 0.17	0.93 ± 0.10		
		CG	1.11 ± 0.18	0.96 ± 0.13		
	Power	EG	1.05 ± 0.13	0.83 ± 0.05		EG < CG ***
		CG	1.11 ± 0.12	0.94 ± 0.05		
Elastic–ML	Normal	EG	0.94 ± 0.15	0.75 ± 0.08	Time *F*(1, 26) = 32.79 *** η^2^p = 0.558	
		CG	0.95 ± 0.25	0.81 ± 0.10		
	Fast	EG	0.87 ± 0.12	0.75 ± 0.17		
		CG	0.88 ± 0.13	0.77 ± 0.12		
	Power	EG	0.80 ± 0.09	0.66 ± 0.05		EG < CG ***
		CG	0.81 ± 0.08	0.76 ± 0.07		
Inelastic–AP	Normal	EG	1.19 ± 0.21	0.97 ± 0.14	G × T *F*(1, 26) = 8.86 ** η^2^p = 0.254	EG < CG *
		CG	1.17 ± 0.15	1.13 ± 0.20		
	Fast	EG	1.14 ± 0.19	0.99 ± 0.14		
		CG	1.08 ± 0.14	1.13 ± 0.24		
	Power	EG	1.07 ± 0.12	0.84 ± 0.06		EG < CG ***
		CG	1.13 ± 0.13	0.95 ± 0.06		
Inelastic–ML	Normal	EG	0.98 ± 0.16	0.73 ± 0.10	G × T *F*(1, 26) = 5.61 * η^2^p = 0.177	EG < CG *
		CG	0.93 ± 0.09	0.83 ± 0.11		
	Fast	EG	0.91 ± 0.18	0.73 ± 0.13		
		CG	0.93 ± 0.11	0.79 ± 0.17		
	Power	EG	0.86 ± 0.08	0.67 ± 0.07		EG < CG ***
		CG	0.87 ± 0.09	0.79 ± 0.08		

Note: AP, anterior–posterior; ML, medial–lateral; G × T, Group × Time. Effect cells report the dominant effect for each task/direction; smaller COP values indicate greater stability. * *p* < 0.05, ** *p* < 0.01, *** *p* < 0.001. Degrees of freedom were (1, 26) for group, time, and group × time effects and (2, 52) for speed and speed × time effects; η^2^p, partial eta-squared.

**Table 5:** Summary of two-way (group × time) and three-way (group × time × speed) ANOVA for surface EMG (%RVC) of the seven trunk and lower-limb muscles under the inelastic and elastic barbell tasks.

Muscle	Barbell	Speed	Group × Time	Speed × Time
ST	Inelastic	11.60 *** (0.481)	4.77 * (0.155)	5.29 * (0.297)
	Elastic	12.19 *** (0.494)	4.36 * (0.144)	n.s.
BF	Inelastic	n.s.	n.s.	n.s.
	Elastic	n.s.	n.s.	n.s.
GM	Inelastic	14.00 *** (0.528)	n.s.	3.73 *
	Elastic	13.82 *** (0.525)	n.s.	3.77 *
Rt. ES	Inelastic	7.68 ** (0.381)	n.s.	4.50 *
	Elastic	22.59 *** (0.644)	n.s.	n.s.
Lt. ES	Inelastic	7.28 ** (0.368)	n.s.	7.07 ** (0.361)
	Elastic	11.72 *** (0.484)	n.s.	n.s.
VL	Inelastic	23.86 *** (0.656)	n.s.	n.s.
	Elastic	43.93 *** (0.778)	n.s.	n.s.
VM	Inelastic	22.70 *** (0.645)	n.s.	3.91 *
	Elastic	34.32 *** (0.733)	n.s.	n.s.

Note: Note: Values are *F* statistics; η^2^p is given in parentheses for significant effects. Degrees of freedom were (1, 26) for group × time effects and (2, 52) for speed and speed × time effects (Greenhouse–Geisser correction applied where sphericity was violated). ST, semitendinosus; BF, biceps femoris; GM, gluteus medius; Rt. ES, right erector spinae; Lt. ES, left erector spinae; VL, vastus lateralis; VM, vastus medialis; n.s., not significant. * *p* < 0.05, ** *p* < 0.01, *** *p* < 0.001.

## Data Availability

The data presented in this study are available upon reasonable request from the corresponding author.
